# Genome-Wide Identification and Characterization of the *VOZ* Gene Family in *Gossypium hirsutum* L. and Functional Characterization in Abiotic Stress and Somatic Embryogenesis

**DOI:** 10.3390/ijms262210965

**Published:** 2025-11-12

**Authors:** Mengmeng Jiang, Conghua Feng, Junbo Zhen, Linlin Liu, Di Liu, Shuling Zhang, Jina Chi

**Affiliations:** 1 Genetics Laboratory, College of Life Science, Hebei University, Baoding 071002, China; jmm18270464849@163.com; 2 Institute of Cotton, Hebei Academy of Agriculture and Forestry Sciences/Key Laboratory of Cotton Biology and Genetic Breeding in Huanghuaihai Semiarid Area, Ministry of Agriculture and Rural Affairs, Shijiazhuang 050051, China; fch19942024@163.com (C.F.); zjb210@126.com (J.Z.); spp2016@126.com (L.L.); liudijoy@126.com (D.L.)

**Keywords:** *GhVOZ* transcription factors, *Gossypium hirsutum*, genome wide analysis, expression pattern, salt stress

## Abstract

Vascular Plant One-Zinc finger (VOZ) transcription factors are pivotal regulators of plant growth and stress adaptation, yet their functional roles in *Gossypium hirsutum*, a key fiber crop, remain poorly characterized. In this study, we systematically identified six *VOZ* genes in *G. hirsutum* and conducted a comprehensive analysis of their phylogenetic relationships, genomic distribution, promoter architecture, and expression profiles. Phylogenetic classification placed the GhVOZ proteins into three distinct clades, and chromosomal localization revealed that family expansion was likely driven by segmental duplication events. Promoter analysis uncovered an abundance of stress-related cis-regulatory elements, suggesting a potential role in abiotic stress signaling. Consistent with this, expression profiling demonstrated that *GhVOZ1/3*, *GhVOZ2/4/5*, and *GhVOZ6* were specifically induced under drought, salt, and cold stress, respectively, with qRT-PCR further confirming their tissue-specific dynamic regulation under salt treatment. Furthermore, the *GhVOZ* family exhibited stage-specific expression patterns during somatic embryogenesis. *GhVOZ1*, *GhVOZ3*, and *GhVOZ4* were upregulated at the early induction phase, implicating them in the initiation of cell reprogramming. In contrast, *GhVOZ2* and *GhVOZ4* showed sustained expression in embryogenic callus at later stages, suggesting a role in maintaining embryogenic competence, whereas *GhVOZ5*—preferentially expressed in non-embryogenic callus—may act as a repressor of embryogenesis. Synteny analysis further highlighted evolutionary conservation and subgenomic divergence of *VOZ* genes in *G. hirsutum*. Collectively, these findings establish *GhVOZs* as key regulators integrating abiotic stress response and somatic embryogenesis, providing a genetic framework for future functional studies and crop improvement.

## 1. Introduction

Transcription factors (TFs) are the proteins that act as on/off switches for gene expression, therefore regulating their function [1]. The TFs act as a nodal point for balancing the trade-off between growth and defense response; they play critical regulatory roles throughout plant growth and development, including processes such as nutrient absorption, nitrogen (N) uptake, transport, and assimilation, environmental signal transduction, and stress adaptation [2,3,4,5]. Vascular Plant One-Zinc finger (VOZ) transcription factors play a crucial role in plant growth, development, and response to abiotic stresses and pathogen infection [6]. Intriguingly, they are exclusively present in vascular plants and the moss *Physcomitrella patens* [7,8]. VOZ proteins harbor two conserved domains: a zinc-finger domain and a C-terminal NAC domain, along with an N-terminal TR domain, where they participate in regulating diverse biological processes, including flowering time control, photomorphogenesis, and signaling pathways for both abiotic and biotic stresses [9,10,11,12,13,14,15,16,17,18]. *VOZ* TFs play vital roles in plant growth and development. Current research demonstrates that *Arabidopsis thaliana* VOZ (AtVOZ) proteins interact with *phytochrome B* to regulate flowering by modulating the expression of key flowering genes such as *FLOWERING LOCUS T* (*FT*) and *FLOWERING LOCUS C* (*FLC*) [8,9]. Conversely, another study indicates that the *VOZ* gene regulates photoperiodic components like *CONSTANS* (*CO*) to promote flowering in crops [12]. In *Solanum lycopersicum*, *SlVOZ1* directly binds to the promoter of *SQUAMOSA PROMOTER BINDING PROTEIN-LIKE* (*SFT*), a major flowering integrator gene, under drought stress, thereby accelerating flowering [19]. VOZ proteins also serve as key targets for the movement protein (MP) of barley yellow dwarf virus GAV (BYDV-GAV) within the flowering regulatory network. The GAV MP promotes VOZ protein degradation via the 26S proteasome pathway, disrupting flowering and causing phenological delays [20].

*VOZ* TFs play a crucial role in plant responses to both biotic and abiotic stresses. *AtVOZ2* enhances drought tolerance and salt resistance in *A. thaliana* through an abscisic acid (ABA)-dependent pathway [21]. In contrast, the overexpression of *AtVOZ2* in transgenic plants increases their susceptibility to drought and cold stress, while simultaneously enhancing their resistance to the fungal pathogen *Colletotrichum higginsianum* [22]. Both *AtVOZ1* and *AtVOZ2* mitigate heat stress by suppressing the expression of dehydration-responsive element-binding factors DEHYDRATIONRESPONSIVE ELEMENT BINDING FACTOR 2C (*DREB2C*) and *DREB2A* [16,23]. The double mutation of *ATVOZ1*-*VOZ2* in *A. thaliana* demonstrates an enhanced tolerance to both drought and cold stress, while simultaneously exhibiting a diminished resistance to heat stress and pathogens [14,19]. Similar functional divergence occurs in crops: the overexpression of *Glycine max VOZ1* (*GmVOZ1*) in the hairy roots of *G. max* significantly enhances resistance to both drought and salt stress, while its RNAi-mediated silencing increases susceptibility to these stresses [24]. In *Chenopodium quinoa*, transcript levels of *CqVOZ1/4* are downregulated under salt and drought stress, whereas *CqVOZ2/3* are upregulated under low temperature, salt, and drought stress, demonstrating stress-specific induction patterns [25]. In *Oryza sativa*, *OsVOZ1* and *OsVOZ2* act as negative and positive regulators, respectively, against the fungal pathogen *Magnaporthe oryzae* [17]. Despite extensive research on VOZ proteins, there is a noticeable absence of systematic analysis concerning the *VOZ* gene family in polyploid crops, especially in economically significant species *G. hirsutum*.

*G. hirsutum* is recognized as one of the world’s foremost economic crops. It serves not only as the primary provider of natural fiber but also significantly contributes to the production of edible oil, animal feed, and biofuels. Furthermore, *G. hirsutum* serves as a model system for studying polyploid evolution and stress adaptation, providing crucial theoretical insights for crop genetic improvement [26,27]. However, *G. hirsutum* yield and fiber quality are significantly constrained by salinity, drought, and extreme temperatures [27,28,29,30]. In recent years, systematic analyses of stress-related TF families such as *NAC*, *WRKY*, and *MYB* have been conducted in *G. hirsutum*, yet a comprehensive genome-wide characterization of the *VOZ* family is still absent [31,32,33]. The importance of *VOZ* in stress adaptation of *G. hirsutum* has been confirmed, and *GhVOZ1* can regulate sodium ion homeostasis by binding to the promoter of *GhAVP1*, thereby enhancing the salt tolerance of *G. hirsutum* [34]. As an important transcription factor, the *VOZ* gene has been reported to play significant roles in various abiotic stresses such as salt and drought stress; meanwhile, the *VOZ* gene family has been studied in various species such as *C. quinoa*, *S.lycopersicum*, *Triticum aestivum*, and *Cucurbitaceae* species. However, research on this gene family in *G. hirsutum* remains limited [25,35,36,37].

In this study, six *VOZ* genes were successfully identified in *G. hirsutum*, and their basic physicochemical properties, chromosomal distribution, gene structure, conserved motifs, and expression profiles were systematically analyzed. A comprehensive analysis of the *GhVOZ* gene family lays a solid foundation for future functional studies of *VOZ* genes. These findings also provide valuable insights into the molecular mechanisms underlying development and stress resistance in *G. hirsutum* or other crops.

## 2. Results

### 2.1. Identification and Characterization of GhVOZ Gene Family

To identify *VOZ* genes in *G. hirsutum*, the protein sequences of *AtVOZ* from *A. thaliana* were used as queries for a local BLAST (Basic Local Alignment Search Tool) search against the *G. hirsutum* genome. A total of six *VOZ* genes were identified and named *GhVOZ1* to *GhVOZ6* based on their chromosomal positions in ascending order. Basic information of the *GhVOZ* genes, including chromosome location, ORF (open reading frame) length, AA (amino acids) number, MW (molecular weight), pI (isoelectric point), and subcellular localization prediction, was analyzed. As shown in Table 1, the protein lengths of the *VOZ* transcription factor family in *G. hirsutum* ranged from 101 aa (*GhVOZ6*) to 483 aa (*GhVOZ2* and *GhVOZ4*), with corresponding molecular weights ranging from 11.65 kDa (*GhVOZ6*) to 53.80 kDa (*GhVOZ2*). The theoretical isoelectric points of the VOZ proteins varied from 5.30 (*GhVOZ1*) to 9.17 (*GhVOZ6*). Among them, five *GhVOZ* proteins had theoretical pI values less than 7, indicating acidic properties, while only *GhVOZ6* had a theoretical pI greater than 7, indicating basic properties. The predictions regarding subcellular localization indicated that all six GhVOZ proteins were situated within the nucleus. These findings lay a fundamental groundwork for further investigation into the functional mechanisms and biological roles of the *VOZ* gene family in *G. hirsutum*.

### 2.2. The Chromosomal Localization of the GhVOZs

**Figure 1:**
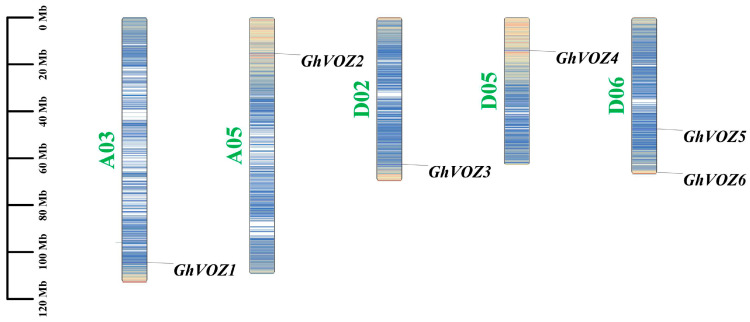
The chromosomal localization of *GhVOZ* genes. Scale: 20 Mb; the bar chart represents the chromosome where the gene is located, with green font indicating the chromosome number. The horizontal lines of different colors on the chromosome represent gene density.

To ascertain the genomic distribution of *GhVOZ* genes, a chromosomal localization map detailing the positions of the *VOZ* gene family members in *G. hirsutum* was constructed. The results showed that the six *VOZ* genes family members are unevenly distributed across five chromosomes of *G. hirsutum* (Figure 1). Specifically, one *VOZ* gene is located on each of the chromosomes A03 (*GhVOZ1*), A05 (*GhVOZ2*), D02 (*GhVOZ3*), and D05 (*GhVOZ4*), while two *VOZ* genes (*GhVOZ5* and *GhVOZ6*) are located on chromosome D06.

### 2.3. Phylogenetic Tree Analysis of the VOZ Genes Across Multiple Species

**Figure 2:**
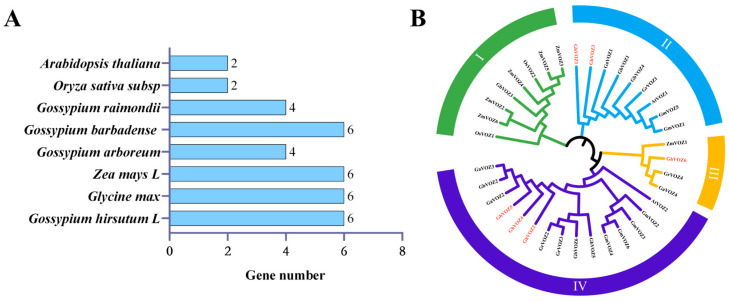
(**A**) Number of *VOZ* genes across *G. hirsutum*, *G. max*, *Z. mays*, *G. arboreum*, *G. barbadense*, *G. raimondii*, *A. thaliana*, and *O. sativa*. (**B**) Phylogenetic analysis of the *VOZ* transcription factors from different species. *GhVOZs* are specifically marked in red.

In order to elucidate the phylogenetic relationships within the *GhVOZ* gene family of *G. hirsutum*, multiple sequence alignment was performed on a total of 36 VOZ proteins, including *G. hirsutum* (6 *GhVOZs*), *G. max* (6 *GmVOZs*), *Zea mays* (6 *ZmVOZs*), *Gossypium arboreum* (4 *GaVOZs*), *Gossypium barbadense* (6 *GbVOZs*), *Gossypium raimondii* (4 *GrVOZs*), *A. thaliana* (2 *AtVOZs*), and *O.sativa* (2 *OsVOZs*) (Figure 2A). A phylogenetic tree was constructed using MEGA (Molecular Evolutionary Genetics Analysis). As shown in Figure 2B, the 36 VOZ proteins were categorized into four distinct groups, labeled as Group I through Group IV. Group I comprises eight members, Group II contains nine, Group III holds four, and Group IV consists of fifteen. Within the GhVOZ proteins, two are classified under Group II, one belongs to Group III, and three are associated with Group IV.

### 2.4. Multiple Sequence Alignment of GhVOZs

**Figure 3:**
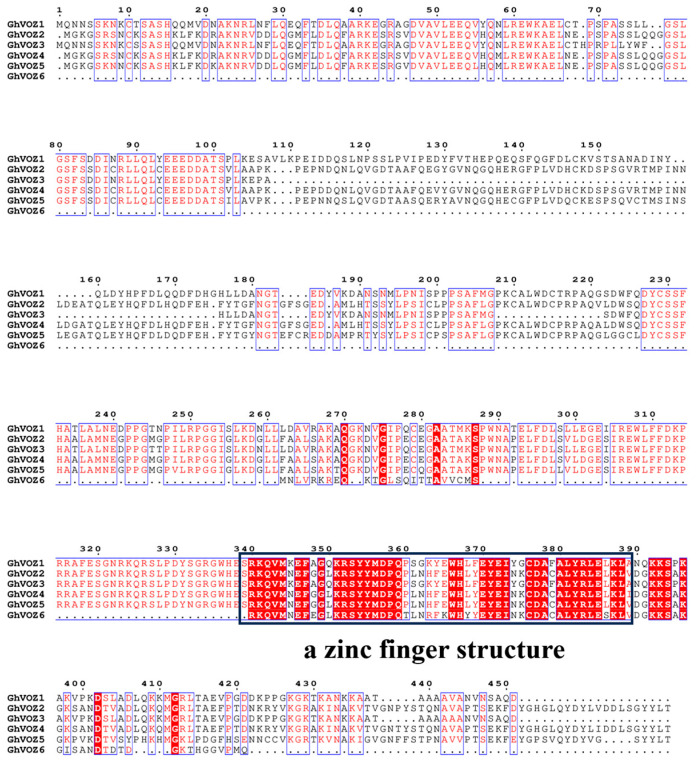
Multiple sequence alignment of *GhVOZs*. Blue boxes represent potentially conserved regions, red text indicates conserved sequences, and red highlighting denotes identical sequences. The black square denotes the conserved C-terminal region, which a zinc finger structure.

The multiple sequence alignment of VOZ proteins from *G. hirsutum* (Figure 3) revealed that a nuclear localization signal and a zinc finger structure were observed in the C-terminal of these VOZ proteins, which may be related to the function of the *VOZ* gene. Notably, the DNA-binding domain is highly conserved in all six *VOZ* members, a feature consistent with their role as transcription factors.

### 2.5. The Phylogenetic Tree Motifs, Conserved Domains, and GENE Structure Analysis of GhVOZ Gene Family

**Figure 4:**
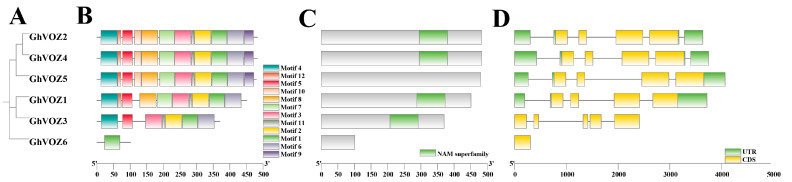
Gene family analysis of *GhVOZs*. (**A**) The phylogenetic tree of GhVOZ proteins. (**B**) The conserved motifs of GhVOZ proteins. Boxes of different colors represent different motifs. (**C**) The conserved domain annotation of GhVOZ proteins. The green boxes represent the *NAM* superfamily. (**D**) Exon–intron structures of the *GhVOZ* genes. The yellow boxes represent the exons, and the green boxes represent the 5′ UTR and the 3′ UTR. Lines represent the introns.

The evolutionary and structural features of the *GhVOZ* gene family were systematically analyzed through phylogenetic reconstruction, motif identification, conserved domain annotation, and exon–intron architecture profiling. Phylogenetic analysis of GhVOZ proteins resolved them into three distinct clades (Groups I–III), with *GhVOZ2*, *GhVOZ4*, and *GhVOZ5* clustering in Group I, *GhVOZ1* and *GhVOZ3* in Group II, and *GhVOZ6* forming Group III (Figure 4A). This clustering pattern correlated with motif composition and gene structure variations. MEME (Multiple Em for Motif Elicitation)-based motif analysis identified 10 conserved motifs, with motif 1 (zinc finger domain) universally present in all GhVOZ proteins, while motifs 2–10 exhibited clade-specific distributions (Figure 4B). For instance, Group I members (*GhVOZ2/4/5*) uniquely retained motifs 5 and 7. Conserved domain annotation via NCBI-CDD (National Center for Biotechnology Information’s Conserved Domain Database) confirmed that the NAC domain (PSSM-ID 460539) present in *GhVOZ1/2/3/4* is notably absent in *GhVOZ5* and *GhVOZ6*, suggesting that the D06 subgeomic genes of this family may have undergone mutations. Gene structure analysis revealed divergent exon–intron organizations across clades. Members of Group I (*GhVOZ2/4/5*) and Group II (*GhVOZ1/3*) exhibited complex structures with 5–7 exons, whereas *GhVOZ6* (Group III) displayed a simplified single-exon architecture (Figure 4C). Additionally, the 5′-UTR regions in *GhVOZ2/4/5* were significantly longer compared to other members, potentially influencing transcriptional regulation (Figure 4D). These structural distinctions align with phylogenetic groupings, underscoring evolutionary conservation and functional diversification within the *GhVOZ* family. Collectively, the integration of phylogenetic, motif, domain, and gene structure analyses provides critical insights into the evolutionary dynamics and functional specialization of *GhVOZ* genes in *G. hirsutum*.

### 2.6. Synteny and Selective Pressure Analysis

**Figure 5:**
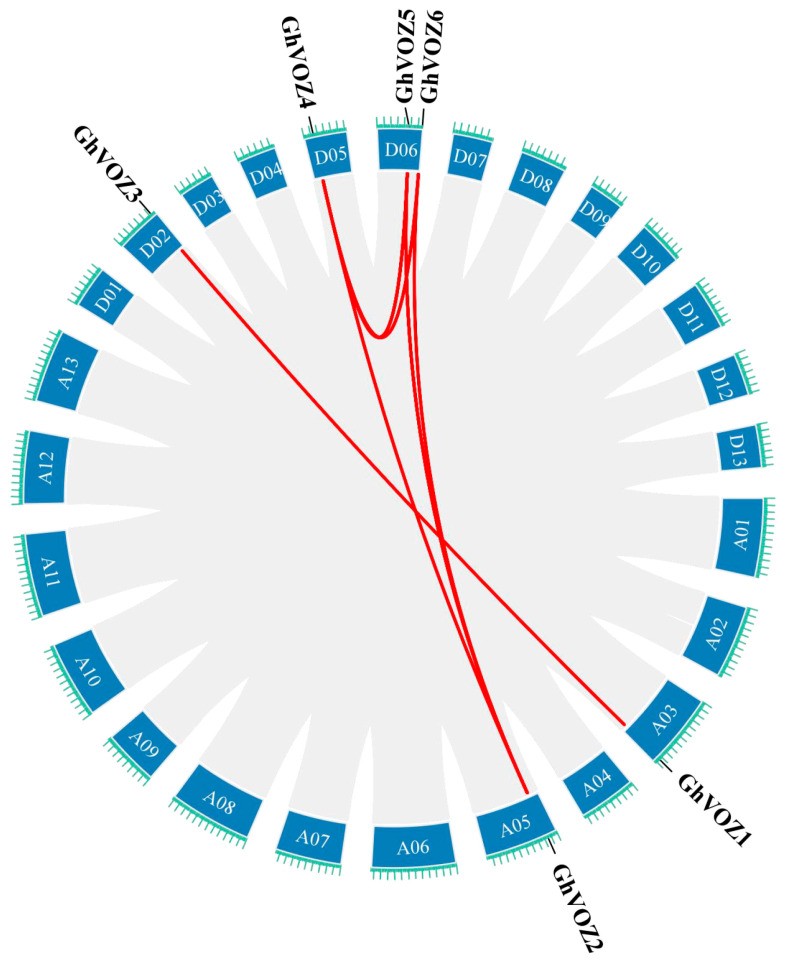
Intraspecific synteny analysis of *GhVOZ* genes. Red lines connect syntenic gene pairs between chromosomes.

To elucidate the gene duplication relationships within the *VOZ* gene family in *G. hirsutum*, intra-genomic synteny analysis of the *VOZ* gene family was conducted using TBtools (Toolkit for Biologists) software (Figure 5). The results revealed eight pairs of syntenic relationships among the six *VOZ* genes, further indicating that the expansion of this gene family primarily resulted from chromosomal segmental duplications. Specifically, no segmental duplications were observed in the A subgenome, while the D subgenome contained three pairs of segmental duplications involving three genes. Between the A and D subgenomes, five pairs of segmental duplications involving six genes were detected. The *G. hirsutum* genome is believed to have formed approximately 10,000 to 20,000 years ago through interspecific hybridization and chromosome doubling between the A genome of *G. arboreum* and the D genome of *G. raimondii*. Therefore, it can be inferred that the expansion of the *G. hirsutum VOZ* gene family via chromosomal segmental duplications occurred after the hybridization of the A and D genomes.

**Figure 6:**
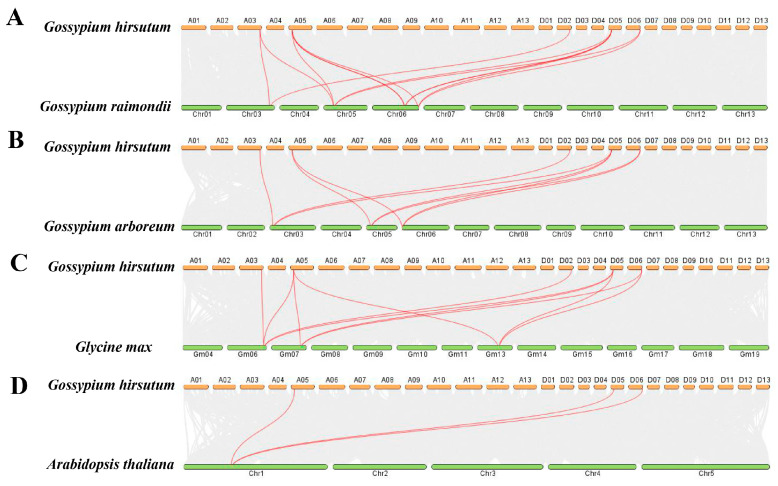
Interspecific collinearity analysis of *GhVOZ* genes. (**A**) Interspecific collinearity analysis between *G. raimondii*, (**B**) Interspecific collinearity analysis between *G. arboreum*, (**C**) Interspecific collinearity analysis between *G. max*, (**D**) Interspecific collinearity analysis between *A. thaliana*. Red lines connect syntenic gene pairs between chromosomes.

This experiment also analyzed the collinearity relationships between the *VOZ* gene family members of *G. hirsutum* and *G. raimondii*, *G. arboreum*, *G. max*, and *A. thaliana*. The results showed that 11 pairs of homologous genes were identified between *G. hirsutum* and *G. raimondii*, involving 5 *GhVOZ* genes and 4 *GrVOZ* genes (Figure 6A); 9 pairs of homologous genes were identified between *G. hirsutum* and *G. arboreum*, involving 5 *GhVOZ* genes and 3 *GaVOZ* genes (Figure 6B); 10 pairs of homologous genes were identified between *G. hirsutum* and *G. max*, involving 5 *GhVOZ* genes and 3 *GmVOZ* genes (Figure 6C); and 3 pairs of homologous genes were identified between *G. hirsutum* and *A. thaliana*, involving 3 *GhVOZ* genes and 1 *AtVOZ* gene (Figure 6D). It can be seen that the conservation between the *VOZ* gene family of *G. hirsutum* and the *VOZ* gene family of *A. thaliana* is relatively low, while the conservation with *G. raimondii*, *G. arboreum*, and *G. max* is higher.

Based on the Ka/Ks (Ka: non-synonymous substitution rate and Ks: synonymous substitution rate) analysis results (Table S2), it is known that the Ka/Ks ratios between the four pairs of paralogous genes within the *G. hirsutum* species are all less than 1. This indicates that the *VOZ* transcription factors in *G. hirsutum* are highly conserved, with limited functional differentiation, and that purifying selection plays a significant role in the molecular evolution of the VOZ transcription factors in *G. hirsutum*.

### 2.7. Analysis of cis-Acting Elements Within the Promoter Region

**Figure 7:**
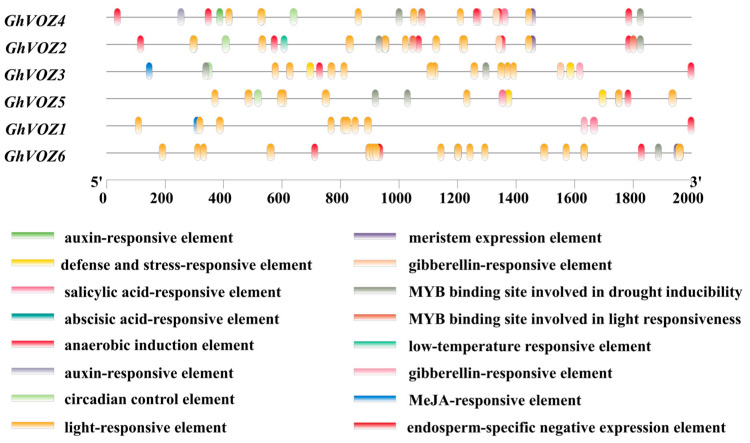
Analysis of cis-acting elements in the promoter region. The boxes of different colors represent different elements.

The promoter analysis of *GhVOZ* genes revealed distinct cis-regulatory architectures correlated with their functional diversification (Figure 7). The promoter region of the *G. hirsutum* VOZ transcription factor gene contains abundant cis-acting elements, the functions of which involve various biological processes, including light response, stress adaptation, hormone signal transduction, and tissue-specific developmental regulation. All *GhVOZ* genes contain light-responsive elements, suggesting that they may be extensively involved in light-regulated transcription processes. In addition, anaerobic induction elements, a type of stress-responsive element, are present in multiple *GhVOZ* genes, indicating that these *VOZ* genes may be involved in hypoxic stress responses; the promoters of *GhVOZ* genes contain a variety of hormone-responsive elements. Among these, abscisic acid (ABA)-responsive elements are significantly enriched in salt-responsive genes (*GhVOZ2/4/5*), suggesting that *VOZ* genes may respond to salt stress through ABA signaling. Furthermore, *GhVOZ6* contains endosperm-specific negative expression elements, suggesting that this gene may have a unique role in seed development.

### 2.8. Gene Expression Analysis

**Figure 8:**
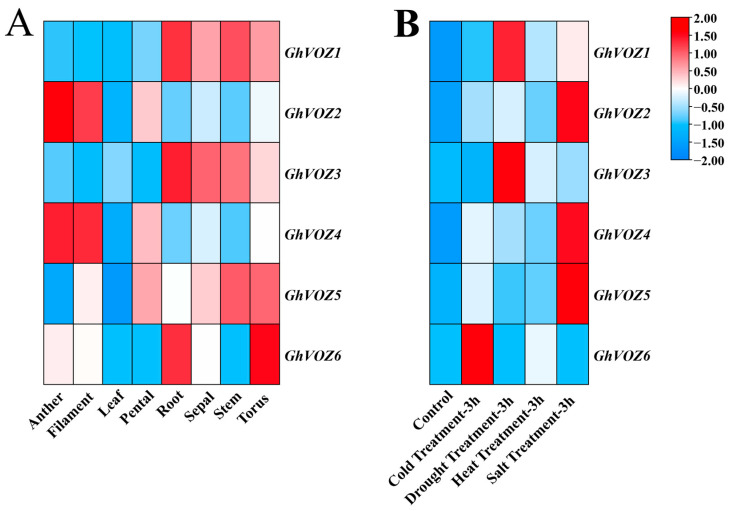
*GhVOZ* gene expression analysis. (**A**) Expression profiles of the *GhVOZ* gene family in various tissues. *X*-axis: Tissue Samples; *Y*-axis: *GhVOZ* Gene names; (**B**) Expression profiles of the *GhVOZ* gene family under various abiotic stresses. *X*-axis: Samples (abiotic stress); *Y*-axis: *GhVOZ* gene names. Color Scale: The color scale utilizes red and blue to denote high and low relative expression levels, respectively.

As shown in Figure 8A, the *GhVOZ* gene family exhibits low expression levels in leaves and petals; *GhVOZ1*, *GhVOZ3*, and *GhVOZ6* show relatively high expression levels in roots, with *GhVOZ6* also displaying elevated expression in the torus. *GhVOZ2* and *GhVOZ4* are primarily expressed in anthers and fibers, while *GhVOZ5* is scarcely expressed in both leaves and anthers. As a multifunctional factor, *VOZ* has also been reported to play critical roles in responding to biotic and abiotic stresses. To further explore the potential functions in resistance to various abiotic stresses, the evaluation of gene expression patterns for *GhVOZs* under conditions of cold, salt, and drought stress was also conducted (Figure 8B). Under different abiotic stress conditions at 3 h, *GhVOZ1* and *GhVOZ3* were significantly upregulated under drought stress, while *GhVOZ2*, *GhVOZ4*, and *GhVOZ5* exhibited significant upregulation under salt stress. *GhVOZ6* was notably upregulated under cold stress, whereas the *GhVOZ* gene family showed no significant expression changes under heat stress compared to the control. The *GhVOZ* gene family members may have distinct roles in pathways that respond to a range of abiotic stresses.

**Figure 9:**
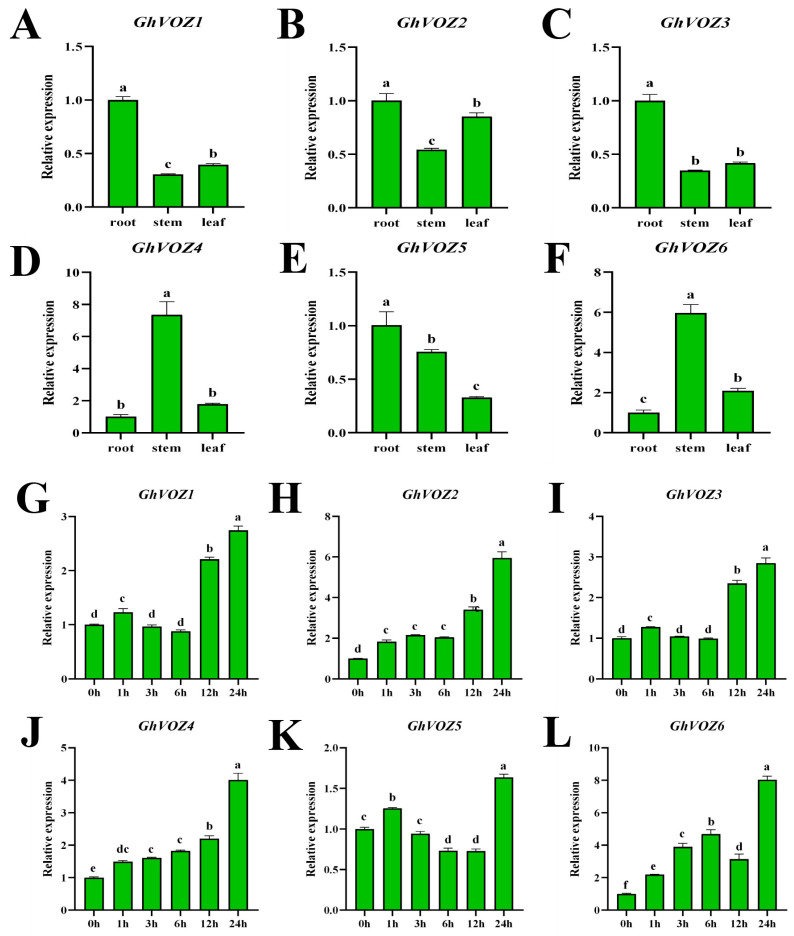
*GhVOZ* gene qRT-PCR analysis. (**A**–**F**) Expression analysis of *GhVOZs* in root, stem, and leaf. In the presented figures, the *Y*-axis delineates the relative expression levels, while the *X*-axis illustrates the samples (tissue). The bars depict the mean values of three biological replicates, accompanied by the standard deviation. (**G**–**L**) Expression analysis of *GhVOZs* under salt stress treatment. The *Y*-axis represents the relative expression levels, and the *X*-axis presents various time points of stress treatments. Bars, again, represent the average values of three biological replicates, along with their standard deviation. Columns marked with distinct letters indicate significant differences at a *p* < 0.05 level, as determined by the Bonferroni test.

Tissue-specific expression analysis can reveal the potential functions of genes during development. Using qRT-PCR, we examined the expression changes in the *GhVOZ* gene family in the roots, stems, and leaves of *G. hirsutum* at the two-leaf and one-bud stage. Using root expression as the control, a bar graph of relative expression levels was plotted. As shown in Figure 9A–F, *GhVOZ1/2/3/5* were predominantly expressed in roots, while *GhVOZ4/6* showed primary expression in stems. The entire *VOZ* family exhibited consistently low expression levels in leaves. To corroborate the involvement of *GhVOZ* genes in salt stress response, *G. hirsutum* seedlings were exposed to a 200 mM NaCl solution. Root was sampled at 0 h, 1 h, 3 h, 6 h, 12 h, and 24 h post-treatment. Using expression levels at 0 h of salt treatment as the control, the bar graph was plotted. As shown in Figure 9G–L, *GhVOZ1-6* exhibited relatively small and inconsistent expression changes from 0 h to 12 h post salt stress. However, at 24 h, all genes showed significant up-regulation compared to 0 h. This suggests that the *VOZ* gene family may have a regulatory role in *G. hirsutum*’s response to salt stress.

### 2.9. GhVOZ Expression Analysis in Callus

To explore the potential roles of *GhVOZ* genes in somatic reprogramming and embryogenic potential establishment, we analyzed their expression patterns during callus induction (7 days, 20 days, 50 days, 80 days) using a real-time fluorescent quantitative method (qRT-PCR) and compared their expressions in EC (embryogenic callus) and NEC (non-embryogenic callus).

**Figure 10:**
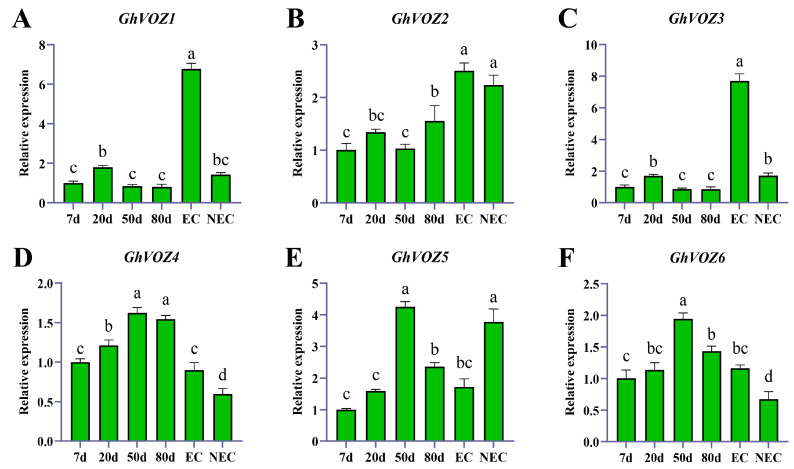
The qRT-PCR analysis of the *GhVOZ* gene in callus. (**A**–**F**) Expression analysis of *GhVOZs* in callus at different stages, EC, and NEC. The *Y*-axis represents the relative expression levels, and the *X*-axis presents various time points of different callus sampled. Bars, again, represent the average values of three biological replicates, along with their standard deviation. Columns marked with distinct letters indicate significant differences at a *p* < 0.05 level, as determined by the Bonferroni test.

The qRT-PCR results using 7 dpi expression as the control, a bar graph of relative expression levels was plotted (Figure 10). At the early stage (7–20 DAI), *GhVOZ1*, *GhVOZ3*, and *GhVOZ4* exhibited significant upregulation, which suggested their potential roles in the initial dedifferentiation process. As the callus entered the proliferation phase (20–50 DAI), the expression of *GhVOZ4*, *GhVOZ5*, and *GhVOZ6* became dominant, indicating that they might be involved in maintaining the growth of a callus. During the late stage (50–80 DAI), which is the key period for obtaining and maintaining embryogenic competence, *GhVOZ2* and *GhVOZ4* showed continuously high expression levels. In contrast, the expression of *GhVOZ5/6* decreased significantly in mature callus.

More obvious differences were observed when comparing EC and NEC. The expression levels of *GhVOZ1/3/4* and *GhVOZ6* were significantly higher in EC than in NEC. This strong association suggests that these four genes might be positive regulators of *G. hirsutum* embryogenic competence. In contrast, *GhVOZ5* showed higher expression in NEC, which suggests it might function as an inhibitor of embryogenesis or is associated with non-embryogenic fate.

## 3. Discussion

VOZ TFs constitute a plant-specific and evolutionarily conserved class of regulators crucial for plant growth, development, and adaptation to abiotic stresses [6]. *G. hirsutum* is an important fiber and oil crop widely planted around the world, which plays an indispensable role in the process of global economic development [38]. Research into the *VOZ* gene family has been conducted in *C. quinoa*, *S.lycopersicum*, *Triticum aestivum*, and *Cucurbitaceae* species; however, studies focusing on this gene family in *G. hirsutum* are limited [25,35,36,37]. Thus, this study aims to conduct a comprehensive genome-wide analysis of *VOZ* genes in *G. hirsutum*.

Gene family expansion, crucial for genome evolution, is mainly driven by tandem and segmental duplication or whole-genome duplication (WGD) [39,40]. Gao et al. deciphered the evolutionary history of the *VOZ* gene family in 46 plant genomes; the *VOZ* gene family provided concise and robust evidence for the establishment of WGD events in the land plant phylogeny [41]. Tandem duplication typically occurs when two or more genes are located on the same chromosome, whereas segmental duplication arises from events between different chromosomes [42]. Firstly, we identified six *VOZ* genes from the genome of *G. hirsutum*. The chromosomal location (Figure 1) and intraspecific collinearity (Figure 5) in *G. hirsutum* indicated that the *VOZ* family in *G. hirsutum* lacked tandem duplication, and the evolution and gene amplification of the *GhVOZ* gene family are mainly derived from segmental duplication events. Collinearity analysis among species (Figure 6) displays the *GhVOZ* gene family evolution from the side. The large number of homologous gene pairs between *G. hirsutum* and its diploid ancestors, *G. arboreum* (A genome) and *G. raimondii* (D genome), suggests that *VOZ* families are evolutionarily conserved within the *Gossypium* genus. This pattern is consistent with the allotetraploid origin of *G. hirsutum* from hybridization of A- and D-genome diploid ancestors [43]. The lower number of homologous pairs with the more distantly related *A. thaliana* reflects the increasing divergence of genome structure over evolutionary time [44]. Moreover, the existence of the homology relationship with *G. max* (another paleopolyploid) suggests that some *VOZ* genes may derive from ancient duplication events shared among angiosperms [41]. Meanwhile, the Ka/Ks ratios for all the homologous pairs of *GhVOZs* are less than 1, indicating that the *VOZ* genes are under purifying selection [45].

Phylogenetic analysis divided the 36 VOZ proteins of eight plant species into four groups (Figure 2A), among which *GhVOZs* were distributed in Groups II–IV. It is worth noting that Group IV members (*GhVOZ2*, *GhVOZ4*, *GhVOZ5*) were preferentially induced under salt stress, while Group II (*GhVOZ1*, *GhVOZ3*) and Group III (*GhVOZ6*) were associated with drought and cold stress response (Figure 8B), respectively; *VOZ* genes might have undergone functional divergence as a result of structural changes during the course of evolution.

The classification of the *NAC* family is mainly based on the sequence similarity and functionality of its N-terminal NAC domain [46]. Ooka et al. firstly made a comprehensive analysis of *NAC* family genes in *O. sativa* and *A. thaliana*, and divided the known *NAC* family gene into two major groups and 18 subgroups by sequence similarity, and the *VOZ* genes of *A. thaliana* were classified into the NAM subgroup in the I big group [47]. However, the NAM domain is conserved in *GhVOZ1*, *GhVOZ2*, *GhVOZ3*, and *GhVOZ4*, while it is absent in *GhVOZ5* and *GhVOZ6* (Figure 4C), which are both localized on chromosome D06; the loss of the NAC domain suggests subgenome-specific evolutionary events, and this structural variation is often related to functional divergence of transcription factor families [48,49]. *GhVOZs* C-terminal zinc finger structure (Figure 3) binds to DNA, thereby regulating gene expression at the transcriptional and translational levels. Analysis of the promoter (Figure 7) showed that the upstream region of *GhVOZ* genes had abundant stress response cis elements, such as ABRE and MYB binding sites. These elements play an important role in abiotic stress responses [50,51]. For example, *AtVOZ2* improved salt and drought tolerance of *A. thaliana* by relying on the ABA pathway, and *GmVOZ1* enhanced drought tolerance through the ABA-dependent pathway [21,22,23,24]. Our analysis revealed a significant enrichment of abscisic acid (ABA) response elements—a diverse class of hormone response elements—within the salt-responsive genes *GhVOZ2/4/5*. This finding suggests that the *VOZ* genes respond to salt stress in the environment, possibly through the ABA signaling pathway, which is consistent with previous studies on *AtVOZ* in *A. thaliana* [19]. *GhVOZs* have different expression patterns under salt, drought, and cold stresses; these results (Figure 8 and Figure 9) suggested that *GhVOZ* genes may act through the stress signaling pathway, but also show lineage-specific regulation. The stage-specific expression pattern of the *GhVOZ* gene during callus induction (Figure 10) suggests its critical role in somatic embryogenesis. Currently, little is known about the regulatory networks involved in the transition from non-embryogenic callus (NEC) to somatic embryos during SE processes [52]. Chen YL et al. found that applying monazamide during the callus proliferation phase could increase abscisic acid (ABA) content, thereby inhibiting callus proliferation and promoting the transformation of callus into embryonic callus (ECs), thus advancing the cotton somatic embryogenesis (SE) process [53]. The upregulation of *GhVOZ1*, *GhVOZ3*, and *GhVOZ4* during the early induction phase (7–20 days after inoculation, DAI) indicates their potential involvement in initiating cell reprogramming and dedifferentiation processes. This observation aligns with existing perspectives that somatic embryogenesis in its early stages is typically accompanied by extensive transcriptional reprogramming and activation of stress-related signaling pathways [54]. The sustained high expression of *GhVOZ2* and *GhVOZ4* during later stages (50–80 DAI)—a critical period for acquiring embryogenic competence—and their significant enrichment in ECs suggest that these TFs are regulatory factors promoting somatic embryogenesis, consistent with known markers of embryogenic potential [55,56]. In contrast, the specific enrichment of *GhVOZ5* in NEC makes it a potential inhibitory factor of the embryogenic program. Additionally, numerous ABA-responsive elements were identified within the *VOZ* genes, establishing *GhVOZ* transcription factors as indispensable participants in the complex regulatory network determining cell fate direction in G. hirsutum.

## 4. Materials and Methods

### 4.1. Identification of Members Belonging to the VOZ Gene Family in G. hirsutum

The genome-wide sequence data, GFF (General Feature Format) annotation files of *A. thaliana* (Version number: Athaliana_447_ Araport11) and the protein sequences of two *AtVOZ* transcription factors were extracted from the publicly accessible genomic repository, TAIR (The Arabidopsis Information Resource, https://www.arabidopsis.org/, accessed on 28 November 2024) [57]. The genome-wide sequence data and GFF annotation files of *G. hirsutum* (Version number: TM1_HAU) were downloaded from the Cotton FGD (Cotton Functional Genomic Database, https://cottonfgd.org/, accessed on 29 November 2024) and Cotton MD (Cotton Multiomics Database, https://yanglab.hzau.edu.cn/CottonMD, accessed on 29 November 2024) [58,59]. The BLAST+ package (version 2.13.0+) was executed on a local Linux system (Ubuntu 22.04 LTS) [60]. The G. hirsutum protein sequence database was constructed using the makeblastdb command, followed by aligning two ATVOZ reference sequences against the constructed database to identify the VOZ gene family protein sequences in G. hirsutum.

### 4.2. Physicochemical Properties and Subcellular Localization Prediction of GhVOZ

The pI, MW, and number of AA of the *GhVOZs* were computed by the pI/MW tool from ExPasy (Expert Protein Analysis System, https://www.expasy.org, accessed on 3 December 2024) [61]. Subcellular localization prediction was performed in the Cell-PLoc (website http://www.csbio.sjtu.edu.cn/bioinf/Cell-PLoc/, accessed on 3 December 2024) [62].

### 4.3. The Chromosomal Localization of the VOZ Gene Family in G. hirsutum

The locational data for all *VOZ* genes within the *G. hirsutum* genome can be procured from the relevant GFF. The *VOZ* genes were subsequently mapped to their respective chromosomes and visualized utilizing the Gene Location Visualize from the GTF/GFF program within the TBtools (version v2.345) [63]. Finally, the resulting images were enhanced and refined using PowerPoint (Microsoft PowerPoint 2021, 64-bit) to improve their aesthetic quality and presentation.

### 4.4. Phylogenetic Tree Analysis

We downloaded the VOZ protein sequences of *G. barbadense*, *O. sativa*, *G. max*, and other plants from the PlantTFDB (Plant Transcription Factor Database, https://planttfdb.gao-lab.org/, accessed on 30 December 2024) [64]. Using the ClustalW default parameters in the MEGA (version 5.05) software [65], we performed multiple sequence alignment on a total of 36 *VOZ* genes family protein sequences derived from *A. thaliana*, *O. sativa*, *G. hirsutum*, and other species. Subsequently, we employed the NJ (Neighbor-Joining) method to construct a phylogenetic tree, and the tree was visually refined using the Evolview online tool (https://www.evolgenius.info/evolview/, accessed on 10 February 2025) [66].

### 4.5. Multiple Sequence Alignment

Conduct a multiple sequence alignment of the GhVOZ protein utilizing ClustalW within the MEGA software, and visualize the sequence alignment results and protein structures using the ESPript3 website (https://espript.ibcp.fr/ESPript/ESPript/index.php, accessed on 27 July 2025) [67].

### 4.6. Gene Family Analysis (Phylogenetic Tree, Motifs, Conserved Domains, Gene Structure)

Submit the identified protein sequences of the *GhVOZ* gene family to the MEGA software for multiple sequence alignment using ClustalW and construct a phylogenetic tree using the NJ method. Subsequently, submit the protein sequences to the MEME (https://meme-suite.org/meme/tools/meme, accessed on 30 December 2024) for conserved motif analysis and to the NCBI-CDD (https://www.ncbi.nlm.nih.gov/Structure/cdd/wrpsb.cgi, accessed on 30 December 2024) for conserved domain analysis [68,69]. Finally, integrate the phylogenetic tree, motif analysis, conserved domain annotation, and GFF file into the Gene Structure View program in TBtools for comprehensive visualization. This approach enables a detailed analysis of the gene family’s evolutionary relationships, functional motifs, structural domains, and exon–intron organization.

### 4.7. Synteny Analysis and Selective Pressure Analysis

Based on the whole-genome sequence and GFF of *G. hirsutum*, an intra-genomic synteny analysis of the *VOZ* gene family was conducted utilizing the MCScanX program within the TBtools software, and the results of the synteny analysis were visualized using the Circos program to generate a circos plot.

Download the genome-wide sequence data, GFF annotation files of *G. raimondii* (Version number: D5_HAU), *Gossypium arboreum* (Version number: A2_HAU) and *G. max* (Version number: Gmax_508_v4.0) from Phytozome (The Plant Comparative Genomics portal of the Department of Energy’s Joint Genome Institute, https://phytozome-next.jgi.doe.gov/, accessed on 15 September 2025) and then upload the genome and GFF files of both *G. hirsutum* and *A. thaliana* or *G. raimondii*, *G. arboretum*, *G. max* into the TBtools One Step MCScanX-Super Fast program for an interspecies synteny analysis [70]. Utilize the Dual Systeny Plot program for visualization purposes.

Based on the CDS (Coding DNA sequence) sequences, protein sequences, and synteny analysis results of the *VOZ* gene family in *G. hirsutum*, the Simple Ka/Ks Calculator integrated within the TBtools was employed to determine the ratio of Ka to Ks, denoted as Ka/Ks, among the members of the *VOZ* gene family. This ratio served as a criterion for identifying the existence of selective pressure. Specifically, a Ka/Ks value less than 1 indicates purifying selection; a Ka/Ks value equal to 1 indicates neutral selection; and a Ka/Ks value greater than 1 indicates positive selection [45].

### 4.8. Analysis of cis-Acting Elements in the Promoter Region

Based on the whole-genome annotation information and whole-genome sequence of *G. hirsutum*, the TBtools software was utilized to extract the upstream 1–2000 bp region of the CDS of the *VOZ* gene family as promoter regions for analysis. The promoter upstream sequences of the *VOZ* gene family extracted from *G. hirsutum* were submitted to PlantCARE (Plant Cis-Acting Regulatory Element, https://bioinformatics.psb.ugent.be/webtools/plantcare/html/, accessed on 10 February 2025) for cis-acting elements analysis [71]. The obtained results were further screened, and finally, the Baisc BioSequese View program in TBtools software was used for corresponding visualization.

### 4.9. Gene Expression Heatmap

Gene expression profiles of *G. hirsutum* in different tissues and after various abiotic stress treatments for 3 h were obtained from CottonFGD. These expression levels were visualized by generating a heatmap using the TBtools HeatMap program.

### 4.10. Plant Materials and Treatments

The *G. hirsutum* material HX1 used in this experiment is all from the Cotton Research Institute of Hebei Academy of Agriculture and Forestry Sciences.

For salt stress treatment, *G. hirsutum* plants were grown in a hydroponic system under a regulated photoperiod, with 16 h of light and 8 h of darkness, at a constant temperature of 25 °C. This environment was maintained until the plants reached the two-leaf and one-bud stage. Samples of roots, stems, and leaves were obtained from the plants, immediately flash-frozen in liquid nitrogen, and subsequently stored in an ultra-low temperature refrigerator at −80 °C. *G. hirsutum* seedlings were subjected to treatment with 200 mM saline solution, and samples were collected 0 h, 1 h, 3 h, 6 h, 9 h, 12 h, and 24 h after treatment, immediately snap-frozen in liquid nitrogen, and subsequently stored in an ultra-low temperature refrigerator.

To induce callus tissue, *G. hirsutum* seeds were sterilized with 0.1% mercuric chloride for 8 min after removing the seed coat, then washed with ddH_2_O 3 times, and inserted into the prepared 1/2 MS medium for cultivation (26 °C, 24 h dark) for 7 days. After 7 days, the hypocotyls were cut into segments of 0.5–1 cm and placed into the callus induction medium for induction. Samples were taken at 7, 20, 50, and 80 days, and continued to be cultured until non-embryogenic and embryogenic callus tissues were obtained. All operations were performed in an ultra-clean workbench.

### 4.11. RNA Extraction and qRT-PCR Analysis

The aforementioned samples were rapidly ground into a fine powder, and total RNA was extracted from each sample utilizing Total RNA Extraction Kit R1200-50T (Solarbio Science & Technology Co., Ltd., Beijing, China) in accordance with the manufacturer’s instructions. The RNA concentration and quality were assessed utilizing the NanoDrop One Quick Start Guide (Thermo Fisher Scientific, Waltham, MA, USA). The process of transcribing RNA into cDNA was executed utilizing the TransScript One-Step gDNA Removal and cDNA Synthesis SuperMix AT311 (TransGen, Beijing, China), following the manufacturer’s prescribed protocol. The relative expression levels of the *GhVOZ* gene family across different tissues and under diverse abiotic stress treatments were determined through qRT-PCR, with the *UBQ7* (*Ghir_D12G021700*) gene serving as an internal control.

The obtained cDNA was mixed with PerfectStart Visual Green qPCR SuperMix AQ621 (Transgen, Beijing, China) to prepare a pre-mixed solution. The qRT-PCR program was executed on the CFX96 Real-Time System (Bio-Rad, Hercules, CA, USA): 95 °C for 30 s; 40 cycles of 95 °C for 5 s and 56 °C for 30 s, and 72 °C for 30 s. The internal reference gene *UBQ7* was selected. Relative expression was quantified using the 2^−ΔΔCt^ method, incorporating four biological replicates and three technical replicates to ensure accuracy. All the primers for qRT-PCR were designed utilizing Primer Premier (version 5.00). A comprehensive list of these primer sequences is provided in Table S1.

## 5. Conclusions

We report a comprehensive genomic and functional characterization of the *VOZ* transcription factor family in *G. hirsutum*, identifying six *GhVOZ* genes and delineating their evolutionary dynamics, structural characteristics, and stress-responsive expression. Phylogenetic reconstruction segregated *GhVOZs* into three clades (Groups II–IV), with relationships strongly supported by conserved motifs and exon–intron structures. Their asymmetric distribution across five chromosomes was primarily facilitated by segmental duplications, highlighting a key evolutionary expansion mechanism. The evolutionary process of this family was subjected to purifying selection, indicating that the functional characteristics of this gene family have remained relatively conserved. Synteny analysis further revealed evolutionary conservation across *G. hirsutum* subgenomes and divergence from other species. While a zinc finger motif was universally conserved, clade-specific NAC domains in *GhVOZ1/2/3/4* suggest neofunctionalization. Promoter analysis uncovered a prevalence of stress-related cis-elements (e.g., ABRE, MYB), implicating these genes in abiotic stress signaling. Expression analysis demonstrated distinct stress induction patterns: *GhVOZ1/3* by drought, *GhVOZ2/4/5* by salt, and *GhVOZ6* by cold, with qRT-PCR validating their dynamic induction in various tissues under salt stress. These findings pinpoint *GhVOZ* TFs as key regulators within the stress response network. This study has for the first time revealed the unique dynamic expression patterns of VOZ gene family members during somatic embryogenesis in *G. hirsutum*, suggesting their crucial role in this process. *GhVOZ1/3/4* may participate in initiating cellular reprogramming, whilst the sustained expression of *GhVOZ2/4* in embryogenised callus and the specific enrichment of *GhVOZ5* in non-embryogenised callus respectively indicate that they may act as initiators and suppressors of embryogenic potential. This demonstrates their integral role in the cell fate regulatory network of *G. hirsutum*. This finding not only expands our understanding of the biological functions of the *VOZ* gene but also provides new insights into overcoming the technical bottleneck of low embryogenesis efficiency in cotton genetic transformation. Meanwhile, the *GhVOZ* gene may integrate diverse hormone signaling pathways such as ABA, forming a sophisticated stress response regulatory network. This provides a solid theoretical foundation and valuable genetic resources for utilizing key *GhVOZ* genes (e.g., salt stress-responsive *GhVOZ2/4/5*) in molecular breeding of stress-resistant cotton cultivars.

## Figures and Tables

**Table 1:** The information on *GhVOZ* genes.

Gene Name	Gene ID	Chr	Genomic Location	ORF	AA	MW (kDa)	PI	Subcellular Localization
*GhVOZ1*	*Ghir_A03G017120.1*	A03	104471192-104474909	1356	451	50.32	5.30	Nucleus
*GhVOZ2*	*Ghir_A05G016300.1*	A05	15362031-15365668	1452	483	53.80	5.54	Nucleus
*GhVOZ3*	*Ghir_D02G018400.1*	D02	62575819-62578229	1113	370	41.44	6.63	Nucleus
*GhVOZ4*	*Ghir_D05G016130.1*	D05	14056821-14060572	1452	483	53.77	5.53	Nucleus
*GhVOZ5*	*Ghir_D06G014660.1*	D06	47534703-47538771	1443	480	53.40	5.81	Nucleus
*GhVOZ6*	*Ghir_D06G022660.1*	D06	66000854-66001159	306	101	11.65	9.17	Nucleus

## Data Availability

The original contributions presented in this study are included in the article/Supplementary Material. Further inquiries can be directed to the corresponding authors.

## Supplementary Materials

The following supporting information can be downloaded at: https://www.mdpi.com/article/10.3390/ijms262210965/s1.

## References

[1] Kumari P., Ojha R., Varshney V., Gupta V., Salvi P., Singh K., Kaur R., Deshmukh R. (2024). Transcription Factors and Their Regulatory Role in Plant Defence Response. Biotechnological Advances for Disease Tolerance in Plants.

[2] Mushineni A., Aundy K., Neelam S., Prakash G., Robin G., Gopala K.S., Ramcharan B. (2020). Black pepper (*Piper nigrum* L.) associated endophytic *Pseudomonas putida BP25* alters root phenotype and induces defense in rice (*Oryza sativa* L.) against blast disease incited by *Magnaporthe oryzae*. Biol. Control.

[3] Guo P., Cui C., Quan B., Zhao J., Ru J., Wang Y., Song Z., Lin A., He S., Wang G. (2025). Identification of the *Q-type ZFP* gene family in *Triticeaes* and drought stress expression analysis in common wheat. Genetica.

[4] Zhang S., Li G., Wang Y., Anwar A., He B., Zhang J., Chen C., Hao Y., Chen R., Song S. (2023). Genome-wide identification of *BcGRF* genes in flowering Chinese cabbage and preliminary functional analysis of *BcGRF8* in nitrogen metabolism. Front Plant Sci..

[5] Zhang J., Zhang X., Liu M., Jin Y., Pai Q., Wu X., Sun D. (2025). Genome-Wide Identification of the HD-ZIP Transcription Factor Family in *Maize* and Functional Analysis of the Role of *ZmHD-ZIP23* in Seed Size. Plants.

[6] He P., Kuai P., Lou Y. (2025). Knocking out a wound-induced vascular plant one-zinc finger gene enhances plant defense against phloem-feeding herbivores. Pest Manag. Sci..

[7] Mitsuda N., Hisabori T., Takeyasu K., Sato M.H. (2004). *VOZ*; Isolation and characterization of novel Vascular plant transcription factors with a One-Zinc Finger from *Arabidopsis thaliana*. Plant Cell Physiol..

[8] Celesnik H., Ali G.S., Robison F.M., Reddy A.S. (2013). *Arabidopsis thaliana* VOZ (Vascular plant One-Zinc finger) transcription factors are required for proper regulation of flowering time. Biol. Open.

[9] Yu R., Jin Y., Liu L., Zhang Y., Wu X., Zuo Y., Qi Y., Yang Z., Zhou J., Xu M. (2024). Potato β-aminobutyric acid receptor *IBI1* manipulates VOZ1 and VOZ2 transcription factor activity to promote disease resistance. Plant Physiol..

[10] Prasad K.V.S.K., Xing D., Reddy A.S.N. (2018). Vascular Plant One-Zinc-Finger (VOZ) transcription factors are positive regulators of salt tolerance in *Arabidopsis*. Int. J. Mol. Sci..

[11] Qu L., Zhong M., Duan F., Li X., Yang J., Zhou Q., Tang D., He R., Liu X., Zhao X. (2024). The *PHYB*-*FOF2*-*VOZ2* module functions to fine-tune flowering in response to changes in light quality by modulating *FLC* expression in *Arabidopsis*. Plant Commun..

[12] Kumar S., Choudhary P., Gupta S., Nath U. (2018). *VASCULAR PLANT ONE-ZINC FINGER1* (*VOZ1*) and *VOZ2* interact with *CONSTANS* and promote photoperiodic flowering transition. Plant Physiol..

[13] Hasan N., Tokuhara N., Noda T., Kotoda N. (2023). Molecular characterization of *Satsuma mandarin* (*Citrus unshiu Marc.*) *VASCULAR PLANT ONE-ZINC FINGER2* (*CuVOZ2*) interacting with *CuFT1* and *CuFT3*. Plant Biotechnol..

[14] Nakai Y., Nakahira Y., Sumida H., Takebayashi K., Nagasawa Y., Yamasaki K., Akiyama M., Ohme-Takagi M., Fujiwara S., Shiina T. (2013). Vascular plant one-zinc-finger protein 1/2 transcription factors regulate abiotic and biotic stress responses in *Arabidopsis*. Plant J..

[15] Wang K., Li C., Cao S., Lei C., Ji N., Zou Y., Tan M., Wang J., Zheng Y., Gao H. (2025). VOZ-dependent priming of salicylic acid-dependent defense against *Rhizopus stolonifer* by β-aminobutyric acid requires the TCP protein TCP2 in peach fruit. Plant J..

[16] Song C., Lee J., Kim T., Hong J.C., Lim C.O. (2018). *VOZ1*, a transcriptional repressor of *DREB2C*, mediates heat stress responses in *Arabidopsis*. Planta.

[17] Wang J., Wang R., Fang H., Zhang C., Zhang F., Hao Z., You X., Shi X., Park C.H., Hua K. (2021). Two VOZ transcription factors link an E3 ligase and an *NLR* immune receptor to modulate immunity in rice. Mol. Plant.

[18] Ganie S.A., Ahammed G.J., Wani S.H. (2020). Vascular plant one zincfinger (VOZ) transcription factors: Novel regulators of abiotic stress tolerance in rice (*Oryza sativa* L.). Genet. Resour. Crop Evol..

[19] Chong L., Xu R., Huang P., Guo P., Zhu M., Du H., Sun X., Ku L., Zhu J.K., Zhu Y. (2022). The tomato *OST1*-*VOZ1* module regulates drought-mediated flowering. Plant Cell.

[20] Zhang Y., Huang C., Zeng Q., Yang M., Wu Y., Tao Y., Ahanger S.A., Rafiq H., Wu Y., Hao X. (2025). Interaction between barley yellow dwarf virus-GAV movement protein and VOZ proteins delays flowering of plant. Mol. Plant Microbe. Interact..

[21] Jensen M.K., Kjaersgaard T., Nielsen M.M., Galberg P., Petersen K., O’Shea C., Skriver K. (2010). The *Arabidopsis thaliana* NAC transcription factor family: Structure-function relationships and determinants of *ANAC019* stress signalling. Biochem. J..

[22] Nakai Y., Fujiwara S., Kubo Y., Sato M.H. (2013). Overexpression of *VOZ2* confers biotic stress tolerance but decreases abiotic stress resistance in *Arabidopsis*. Plant Signal Behav..

[23] Koguchi M., Yamasaki K., Hirano T., Sato M.H. (2017). Vascular plant one-zinc-finger protein 2 is localized both to the nucleus and stress granules under heat stress in *Arabidopsis*. Plant Signal Behav..

[24] Li B., Zheng J.C., Wang T.T., Min D.H., Wei W.L., Chen J., Zhou Y.B., Chen M., Xu Z.S., Ma Y.Z. (2020). Expression analyses of soybean VOZ transcription factors and the role of *GmVOZ1G* in drought and salt stress tolerance. Int. J. Mol. Sci..

[25] Shi P., Jiang R., Li B., Wang D., Fang D., Yin M., Yin M., Gu M. (2022). Genome-Wide analysis and expression profiles of the *VOZ* gene family in quinoa (*Chenopodium quinoa*). Genes.

[26] Wang M., Tu L., Yuan D., Wang M., Tu L., Yuan D., Zhu D., Shen C., Li J., Liu F. (2019). Reference genome sequences of two cultivated allotetraploid cottons, *Gossypium hirsutum* and *Gossypium barbadense*. Nat. Genet..

[27] Chang H., Jiang M., Chu X., Jing Y., Wei J., Zhang G., Yan Y., Du X., Li Z. (2025). The molecular basis for cotton seedling response to salt stress based on genome-wide association study and transcriptome analysis. Theor. Appl. Genet..

[28] Huan T., Zhang X., Lv M., Zhou H., Zhao Y., Yu D., Sun Y. (2025). Phylogeny-based comparative analysis of gene expression modulation upon drought stress across three cotton diploids. BMC Plant Biol..

[29] Mehmood M., Tanveer N.A., Joyia F.A., Ullah I., Mohamed H.I. (2025). Effect of high temperature on pollen grains and yield in economically important crops: A review. Planta.

[30] Qiao M., Hong C., Jiao Y., Hou S., Gao H. (2024). Impacts of Drought on Photosynthesis in Major Food Crops and the Related Mechanisms of Plant Responses to Drought. Plants.

[31] Bai S., Niu Q., Wu Y., Xu K., Miao M., Mei J. (2022). Genome-Wide Identification of the NAC Transcription Factors in *Gossypium hirsutum* and Analysis of Their Responses to *Verticillium wilt*. Plants.

[32] Dong T., Su J., Li H., Du Y., Wang Y., Chen P., Duan H. (2024). Genome-Wide Identification of the *WRKY* Gene Family in Four Cotton Varieties and the Positive Role of *GhWRKY31* in Response to Salt and Drought Stress. Plants.

[33] Su J., Zhan N., Cheng X., Song S., Dong T., Ge X., Duan H. (2024). Genome-wide analysis of cotton MYB transcription factors and the functional validation of *GhMYB* in response to drought stress. Plant Cell Physiol..

[34] Lian B., Wu A., Wu H., Lv X., Sun M., Li Y., Lu Z., Li S., An L., Guo X. (2024). *GhVOZ1*-*AVP1* module positively regulates salt tolerance in *G. hirsutum* (*Gossypium hirsutum* L.). Int. J. Biol. Macromol..

[35] Uluisik S., Kiyak A., Kurt F., Filiz E. (2022). Genome-wide identification of the VOZ transcription factors in tomato (*Solanum lycopersicum*): Their functions during fruit ripening and their responses to salinity stress. J Hortic Sci Biotech..

[36] Sharma Y., Dixit S., Singh K., Upadhyay S.K. (2025). Vascular plant one-zinc finger transcription factors: Exploration of characteristic features, expression, coexpression and interaction suggested their diverse role in bread wheat (*Triticum aestivum* L.). Plant Growth Regul..

[37] Xu M., Zhang Z., Jiao Y., Tu Y., Zhang X. (2024). Genome-wide identification of Vascular Plant One-Zinc-Finger gene family in six *Cucurbitaceae* species and the role of *CmoVOZ2* in salt and drought stress tolerance. Genes.

[38] Abdelraheem A., Esmaeili N., O’Connell M., Zhang J.F. (2019). Progress and perspective on drought and salt stress tolerance in cotton. Ind. Crops Prod..

[39] Shukla R., Pokhriyal E., Das S. (2025). Complex Interplay of Tandem, Segmental, Whole Genome Duplication, and Re-organization Drives Expansion of *SAUR* Gene Family in *Brassicaceae*. Biochem Genet..

[40] Flagel L.E., Wendel J.F. (2009). Gene duplication and evolutionary novelty in plants. New Phytol..

[41] Gao B., Chen M., Li X., Liang Y., Zhu F., Liu T., Zhang D., Wood A.J., Oliver M.J., Zhang J. (2018). Evolution by duplication: Paleopolyploidy events in plants reconstructed by deciphering the evolutionary history of VOZ transcription factors. BMC Plant Biol..

[42] Liu Y., Li D., Liu Y., Wang J., Liu C. (2024). Genome-Wide Identification and Evolution-Profiling Analysis of *TPS* Gene Family in *Triticum* Plants. Int. J. Mol. Sci..

[43] Li J., Yuan D., Wang P., Wang Q., Sun M., Liu Z., Si H., Xu Z., Ma Y., Zhang B. (2021). Cotton pan-genome retrieves the lost sequences and genes during domestication and selection. Genome Biol..

[44] Hu T.T., Pattyn P., Bakker E.G., Cao J., Cheng J.F., Clark R.M., Fahlgren N., Fawcett J.A., Grimwood J., Gundlach H. (2011). The *Arabidopsis lyrata* genome sequence and the basis of rapid genome size change. Nat. Genet..

[45] La H.V., Chu H.D., Tran C.D., Nguyen K.H., Le Q.T.N., Hoang C.M., Cao B.P., Pham A.T.C., Nguyen B.D., Nguyen T.Q. (2022). Insights into the gene and protein structures of the *CaSWEET* family members in chickpea (*Cicer arietinum*), and their gene expression patterns in different organs under various stress and abscisic acid treatments. Gene.

[46] Xiong H., He H., Chang Y., Miao B., Liu Z., Wang Q., Dong F., Xiong L. (2025). Multiple roles of NAC transcription factors in plant development and stress responses. J. Integr. Plant Biol..

[47] Ooka H., Satoh K., Doi K., Nagata T., Otomo Y., Murakami K., Matsubara K., Osato N., Kawai J., Carninci P. (2003). Comprehensive Analysis of *NAC* Family Genes in *Oryza sativa* and *Arabidopsis thaliana*. DNA Res..

[48] Freeling M. (2009). Bias in plant gene content following different sorts of duplication: Tandem, whole-genome, segmental, or by transposition. Annu. Rev. Plant Biol..

[49] Rensing S.A. (2014). Gene duplication as a driver of plant morphogenetic evolution. Curr. Opin. Plant Biol..

[50] Chen Q.S., Liu P.P., Li Z.F., Zheng Q.X., Zhou H.N., Liu J.Y., Cao P.J., Fang M. (2024). Accumulated Endogenous Abscisic Acid Contributes to the Cold Tolerance of Pre-planted *Cultivated tobacco*. Plant Mol. Biol. Rep..

[51] Chen C.Y., Zhao W.J., Li X.L., Wen K., Zi Y.Q., Zhao K., Chen D.M., Zhang H.Y., Liu X.Z. (2025). Genome-wide identification of the MYB transcription factor family in kiwifruit and analysis of its expression pattern in response to salt stress. Acta Physiol. Plant..

[52] Guo H., Guo H., Zhang L., Tang Z., Yu X., Wu J., Zeng F. (2019). Metabolome and Transcriptome Association Analysis Reveals Dynamic Regulation of *Purine Metabolism* and Flavonoid Synthesis in Transdifferentiation during Somatic Embryogenesis in Cotton. Int. J. Mol. Sci..

[53] Chen Y., Yu H., Wang Y., Li F., Xing Y., Ge X. (2022). Uniconazole Augments Abscisic Acid in Promoting Somatic Embryogenesis in Cotton (*Gossypium hirsutum* L.). Front. Plant Sci..

[54] Fehér A. (2019). Callus, Dedifferentiation, Totipotency, Somatic Embryogenesis: What These Terms Mean in the Era of Molecular Plant Biology?. Front Plant Sci..

[55] Yang X., Zhang X., Yuan D., Jin F., Zhang Y., Xu J. (2012). Transcript profiling reveals complex auxin signalling pathway and transcription regulation involved in dedifferentiation and redifferentiation during somatic embryogenesis in cotton. BMC Plant Biol..

[56] Nolan K.E., Irwanto R.R., Rose R.J. (2003). Auxin up-regulates *MtSERK1* expression in both *Medicago truncatula* root-forming and embryogenic cultures. Plant Physiol..

[57] Reiser L., Bakker E., Subramaniam S., Chen X., Sawant S., Khosa K., Prithvi T., Berardini T.Z. (2024). The *Arabidopsis* information resource in 2024. Genetics.

[58] Zhu T., Liang C., Meng Z., Sun G., Meng Z., Guo S., Zhang R. (2017). CottonFGD: An integrated functional genomics database for cotton. BMC Plant Biol..

[59] Yang Z.Q., Wang J., Huang Y.M., Wang S.B., Wei L.L., Liu D.X., Weng T.L., Xiang J.H., Zhu Q., Yang Z.E. (2023). CottonMD: A multi-omics database for cotton biological study. Nucleic Acids Res..

[60] Camacho C., Coulouris G., Avagyan V.M.N., Papadopoulos J., Bealer K., Madden T.L. (2009). BLAST+: Architecture and applications. BMC Bioinf..

[61] Duvaud S., Gabella C., Lisacek F., Stockinger H., Ioannidis V., Durinx C. (2021). Expasy, the Swiss Bioinformatics Resource Portal, as designed by its users. Nucleic Acids Res..

[62] Chou K.C., Shen H.B. (2010). Cell-PLoc 2.0: An improved package of web-servers for predicting subcellular localization of proteins in various organisms. Nat. Sci..

[63] Chen C., Chen H., Zhang Y., Thomas H.R., Frank M.H., He Y., Xia R. (2020). TBtools: An integrative toolkit developed for interactive analyses of big biological data. Mol. Plant.

[64] Jin J.P., Tian F., Yang D.C., Meng Y.Q., Kong L., Luo J., Gao G. (2017). PlantTFDB 4.0: Toward a central hub for transcription factors and regulatory interactions in plants. Nucleic Acids Res..

[65] Tamura K., Peterson D., Peterson N., Stecher G., Nei M., Kumar S. (2011). MEGA5: Molecular evolutionary genetics analysis using maximum likelihood, evolutionary distance, and maximum parsimony methods. Mol. Biol. Evol..

[66] Subramanian B., Gao S., Lercher M.J., Hu S., Chen W.H. (2019). Evolview v3: A webserver for visualization, annotation, and management of phylogenetic trees. Nucleic Acids Res..

[67] Robert X., Guillon C., Gouet P. (2025). FoldScript: A web server for the efficient analysis of AI-generated 3D protein models. Nucleic Acids Res..

[68] Bailey T.L., Johnson J., Grant C.E., Noble W.S. (2015). The MEME Suite. Nucleic Acids Res..

[69] Wang J., Chitsaz F., Derbyshire M.K., Gonzales N.R., Gwadz M., Lu S., Marchler G.H., Song J.S., Thanki N., Yamashita R.A. (2023). The conserved domain database in 2023. Nucleic Acids Res..

[70] Goodstein D.M., Shu S.Q., Howson R., Neupane R., Hayes R.D., Fazo J., Mitros T., Dirks W., Hellsten U., Putnam N. (2012). Phytozome: A comparative platform for green plant genomics. Nucleic Acids Res..

[71] Lescot M., Dhais P., Thijs G., Marchal K., Moreau Y., Van de Peer Y., Rouzé P., Rombauts S. (2002). PlantCARE, a database of plantcis-acting regulatory elements and a portal to tools for in silico analysis of promoter sequences. Nucleic Acids Res..

